# APRI and FIB-4 scores are not associated with neurocognitive impairment in HIV-infected persons

**DOI:** 10.7448/IAS.17.4.19658

**Published:** 2014-11-02

**Authors:** Raffaella Libertone, Pietro Balestra, Patrizia Lorenzini, Carmela Pinnetti, Martina Ricottini, Samanta Menichetti, Maria Maddalena Plazzi, Alberto Giannetti, Valerio Tozzi, Andrea Antinori, Adriana Ammassari

**Affiliations:** Clinical Department, National Institute for Infectious Diseases, Rome, Italy

## Abstract

**Introduction:**

Chronic liver disease leads to neurocognitive impairment (NCI) and more advanced liver fibrosis is associated with greater deficits. Further, cognitive performances do not differ significantly among patients affected by diverse types of chronic liver diseases. Thus, it would be useful to have a clinical tool associated with early cognitive change applicable to the HIV-infected population with high HCV prevalence. Aim of the analysis was to assess the association between NCI and aspartate aminotransferase-platelet ratio index (APRI) or Fibrosis-4, which are non-invasive scores used to assess liver fibrosis.

**Materials and Methods:**

Single-centre, retrospective, cross-sectional analysis of cART-treated HIV-infected patients undergoing neuropsychological assessment (NPA) by a set of 14 standardized and comprehensive tests on five different domains: concentration and speed of mental processing; mental flexibility; memory, fine motor functioning; visual-spatial and constructional abilities. NPA obtained from the same patient were included, if collected while receiving different cART. Patients were classified as having NCI, if they scored >1 SD below the normative mean in at least two tests, or below >2 SD in one test. HIV-associated neurocognitive disorders (HAND) were classified according to Frascati's criteria, controlling for confounding comorbidities. Univariable analysis and multivariable logistic regression models were carried out.

**Results:**

A total of 556 HIV-infected cART-treated patients from 2006 to 2013 were included: male 78%; IVDUs 14%; CDC stage C 31%; median CD4 nadir 200/mm^3^; median current CD4 501/mm^3^; undetectable HIV-RNA in 368 (67%); and HCV-positivity in 150 (29%). Frequency among score levels was for FIB-4: min-1.44=404 (73%), 1.45–3.25=118 (21%), 3.26-max=28 (5%); for APRI: min-0.5=414 (75%), 0.5–1.5=112 (20%), 1.5-max=24 (4%). Median FIB-4 and APRI were 0.98 and 0.27 in HIV+/HCV- and 1.40 and 0.50 in HIV+/HCV+ individuals, respectively. HAND was found in 176 (32%): 91 ANI, 73 MND, 12 HAD. Association of variables with NCI are shown in Table 1.

**Conclusions:**

In this large population, HAND was not associated with commonly used non-invasive liver fibrosis scores. As aetiology of cognitive dysfunction in HIV mono- and HCV co-infected patients is multifactorial and partially unknown, our results support the hypothesis of a direct or indirect effect on cognitive function of the viruses, rather than the consequence of alterations residing outside the brain.

**Table 1:** Association of variables with NCI

	Risk of HAND								
	
	Univariate analysis			Multivariate analysis -1			Multivariate analysis -2		
	
	HR	95% CI	p	HR	95% CI	p	HR	95% CI	p
Years of education (each y more)	0.80	0.76–0.85	<0.01	0.81	0.76–0.87	<0.01	0.81	0.76–0.87	<0.01
BMI <18, each point more	2.69	1.24–5.86	0.01	2.19	0.85–5.66	n.s.	2.08	0.80–5.40	n.s
CDC stage C	2.52	1.65–3.86	<0.01	1.96	1.15–3.36	0.01	1.94	1.14–3.32	0.01
HCV-positivity	2.46	1.67–3.64	<0.01	1.75	1.02–3.00	0.04	1.63	0.95–2.81	n.s.
CD4/mmc at nadir (each 100 cells)	0.79	0.70–0.89	<0.01	0.98	0.83–1.16	n.s.	0.98	0.83–1.17	n.s.
CD4/mmc at NPA (each 100 cells)	0.93	0.87–0.99	0.02	0.94	0.85–1.04	0.19	0.89	0.77–1.03	n.s
e-GFR at CG <60 mL/min	1.92	1.28–2.88	0.002	1.49	0.87–2.56	n.s.	1.49	0.87–2.54	n.s.
FIB-4 score									
Min/1.44	1.00			1.00					
1.45–3.25	1.75	1.16–2.64	0.01	0.94	0.55–1.60	n.s			
3.26-max	3.77	1.71–8.28	<0.01	1.23	0.44–3.41	n.s.			
APRI score									
Min/1.44	1.00						1.00		
1.45–3.25	1.88	1.22–2.90	<0.01				1.37	0.79–2.37	n.s.
3.26-max	2.60	1.14–5.95	0.02				1.35	0.50–3.65	n.s.

